# Oligodendrocyte HCN2 Channels Regulate Myelin Sheath Length

**DOI:** 10.1523/JNEUROSCI.2463-20.2021

**Published:** 2021-09-22

**Authors:** Matthew Swire, Peggy Assinck, Peter A. McNaughton, David A. Lyons, Charles ffrench-Constant, Matthew R. Livesey

**Affiliations:** ^1^Centre for Regenerative Medicine, Institute for Regeneration and Repair, University of Edinburgh, Edinburgh EH16 4UU, United Kingdom; ^2^Centre for Discovery Brain Sciences, University of Edinburgh, Edinburgh EH8 9XD, United Kingdom; ^3^Wolfson Institute for Biomedical Research, University College London, London WC1E 6BT, United Kingdom; ^4^Wolfson Centre for Age-Related Diseases, King's College London, London WC2R 2LS, United Kingdom; ^5^Sheffield Institute for Translational Neuroscience, University of Sheffield, Sheffield S10 2HQ, United Kingdom

**Keywords:** HCN, myelin, myelination, oligodendrocyte, physiology, sheath

## Abstract

Oligodendrocytes generate myelin sheaths vital for the formation, health, and function of the CNS. Myelin sheath length is a key property that determines axonal conduction velocity and is known to be variable across the CNS. Myelin sheath length can be modified by neuronal activity, suggesting that dynamic regulation of sheath length might contribute to the functional plasticity of neural circuits. Although the mechanisms that establish and refine myelin sheath length are important determinants of brain function, our understanding of these remains limited. In recent years, the membranes of myelin sheaths have been increasingly recognized to contain ion channels and transporters that are associated with specific important oligodendrocyte functions, including metabolic support of axons and the regulation of ion homeostasis, but none have been shown to influence sheath architecture. In this study, we determined that hyperpolarization-activated, cyclic nucleotide-gated (HCN) ion channels, typically associated with neuronal and cardiac excitability, regulate myelin sheath length. Using both *in vivo* and *in vitro* approaches, we show that oligodendrocytes abundantly express functional, predominantly HCN2 subunit-containing ion channels. These HCN ion channels retain key pharmacological and biophysical features and regulate the resting membrane potential of myelinating oligodendrocytes. Further, reduction of their function via pharmacological blockade or generation of transgenic mice with two independent oligodendrocyte-specific HCN2 knock-out strategies reduced myelin sheath length. We conclude that HCN2 ion channels are key determinants of myelin sheath length in the CNS.

**SIGNIFICANCE STATEMENT** Myelin sheath length is a critical determinant of axonal conduction velocity, but the signaling mechanisms responsible for determining sheath length are poorly understood. Here we find that oligodendrocytes express functional hyperpolarization-activated, cyclic nucleotide-gated 2 (HCN2) ion channels that regulate the length of myelin sheaths formed by oligodendrocytes in myelinating cultures and in the mouse brain and spinal cord. These results suggest that the regulation of HCN2 channel activity is well placed to refine sheath length and conduction along myelinated axons, providing a potential mechanism for alterations in conduction velocity and circuit function in response to axonal signals such as those generated by increased activity.

## Introduction

Myelin sheaths accelerate axonal action potential conduction velocity through the establishment of saltatory conduction (Huxley and Stampfli, 1949; Rushton, 1951; Waxman, 1997; Cohen et al., 2020). Myelin sheath length is a critical property that directly impacts conduction velocity by influencing the spacing between nodes of Ranvier. Interestingly, sheath lengths vary significantly throughout the CNS and even along the same axon, suggesting that the regulation of the precise length of myelin sheaths might fine-tune signal propagation and circuit synchronization (Pajevic et al., 2014; Fields and Richardson 2019). Despite the importance of myelin sheath length for axonal function, how sheath length is controlled is not well understood.

Recent transcriptomic and proteomic analyses of the oligodendrocyte lineage indicate that mature oligodendrocyte populations express a number of ion channels, transporters, and receptors with currently undefined roles (Zhang et al., 2014; Marques et al., 2016; Thakurela et al., 2016; Suminaite et al., 2019). Notably, such studies have identified that oligodendrocytes express hyperpolarization-activated, cyclic nucleotide-gated (HCN) ion channels. Electrophysiological studies have also demonstrated the presence of membrane potential responses in oligodendrocytes, as would be expected in the presence of HCN channel-mediated currents (Larson et al., 2016). Given that HCN ion channels are known for regulating basal neuronal and cardiac membrane excitability through their hyperpolarization-activated depolarizing currents (Biel et al., 2009) and that oligodendrocytes have a hyperpolarized resting membrane potential (RMP; Larson et al., 2016), we reasoned that HCN ion channels might play a physiological role in oligodendrocytes.

Here, we show that functional HCN2-containing ion channels are expressed in mature oligodendrocytes and contribute to the baseline membrane conductance of mature oligodendrocytes. We further demonstrate, using both pharmacological and conditional knock-out strategies, that HCN2 channel activity regulates myelin sheath length. We propose that oligodendrocyte HCN2 ion channels play an active role in the dynamic refinement of myelin sheath length.

## Materials and Methods

### 

#### 

##### Mice.

Animal husbandry and experiments were performed under UK Home Office project licenses issued under the Animals (Scientific Procedures) Act. *HCN2^flox/flox^* mice, where exons 2 and 3 coding five of the six transmembrane segments are flanked with Cre-LoxP sites (Emery et al., 2011), were crossed to mice expressing Cre recombinase under the control of the PDGFRα promoter obtained from The Jackson Laboratory (catalog #013148) or under the control of the 2′-3′-cyclin nucleotide phosphodiesterase (CNP) gene (Lappe-Siefke et al., 2003). Offspring were then backcrossed to create mice heterozygous for the floxed allele and carriers for the Cre transgene. Experimental mice were obtained by crossing these animals, generating both HCN2 wild-type control (*Hcn2^f/f^*) and HCN2 floxed homozygous mice (*Hcn2^f/f^*; *Pdgfra-cre*^+/−^ or *CNPase-cre*^+/−^) in the same litters. Mice were genotyped by transnetyx and confirmed as a conditional knockout (cKO) by performing immunostaining for HCN2 and electrophysiology (described below). Mice of each genotype were used at the ages and in the numbers stated in the Results.

##### Antibodies.

The following antibodies were obtained commercially: HCN2 (1:1000; catalog #APC-030, Alomone Labs); CNPase (1:2000; catalog #AMAb91072, Atlas); myelin basic protein (MBP; 1:250; catalog #MCA409S, Serotec); Olig2 (1:100; catalog #AB9610, Millipore); CC1 (1:300; catalog #ab16794, Abcam); MAG (1:100; catalog #MAB1567, Sigma-Aldrich); and CASPR (1:100; catalog #ab34151, Abcam). The Sox10 antibody was a gift from M. Wegner (University of Erlangen, Erlangen, Germany (1:5000).

##### Immunofluorescence staining.

Animals were intracardially perfused with 4% PFA (w/v; Sigma-Aldrich) in PBS, after which brains were postfixed overnight and embedded in 2% low-melting point agarose. Using a Leica vibratome, 100 µm coronal free-floating sections were cut. Sections underwent antigen retrieval in 0.05% Tween 20 and 10 mm tri-sodium citrate, pH 6.0 at 95°C for 20 min. Sections were then blocked for 3 h at room temperature in 10% goat serum and 0.25% Triton in PBS. Primary antibodies were incubated in block solution at 4°C on a rocker for 24 h. Sections were washed with PBS for 3 h at room temperature and stained using species-specific Alexa fluorophore-conjugated antibodies in block solution for 4 h at room temperature. Sections were washed in PBS for a further 3 h and stained with Hoechst stain for 20 min and mounted onto slides with Fluoromount-G. Sections were analyzed with the experimenter blind to experimental condition and/or genotype, as below. Oligodendrocyte morphology was assessed as described previously (Swire et al., 2019). Briefly, random areas of the prefrontal cortex layer II/III were imaged at 63× magnification using an SP8 confocal microscope. Seven individual CNP-positive oligodendrocytes per mouse, each with all myelin sheaths present within the 100 µm section as assessed by following each process from the cell body and ensuring none exited the section, were imaged using a *z* step size of 0.5 µm. Analysis was performed blind using an ImageJ plugin, simple neurite tracer (Longair et al., 2011).

To analyze oligodendrocyte number, five fields of 40× magnification were taken from 2 × 50 µm sections per mouse of cortical layer V or spinal cord gray matter using a Zeiss LSM880 airyscan confocal microscope. All CC1/SOX10-positive oligodendrocytes were counted, whether HCN2-positive or HCN2-negative.

##### Teased spinal cord fibers.

CNS tissue was fixed by cardiac perfusion with 4% formaldehyde in PBS. The cervical spinal cord was dissected out and postfixed for 30 min in 4% formaldehyde. The meninges were removed, and the ventral white matter was isolated in cold PBS and cut into pieces ∼2 mm long. Fibers were then teased onto SuperFrost Plus slides using acupuncture needles. Slides were stored at −20°C in airtight containers until immunolabeling. Sections were blocked for 1 h at room temperature in 3% normal donkey serum, 2% bovine serum albumin, and 0.1% Triton X-100 in PBS. Primary antibodies were incubated in block solution at 4°C overnight. Sections were washed with PBS for 3× 15 min at room temperature and stained using species-specific Alexa fluorophore-conjugated antibodies in block solution for 1 h at room temperature. Sections were washed in PBS for a further 3× 15 min and stained with Hoechst stain for 5 min and mounted onto slides with Fluoromount-G. Tiled *z*-stacks were acquired using a Leica SP8 confocal microscope. Maximum intensity projection images of *z*-stacks were used for analysis. Complete myelin sheaths were determined by two CASPR-positive paranodes connected by a continuous MAG-positive myelin sheath. Images were processed and analyzed using ImageJ.

##### Rat oligodendrocyte precursor cell culture.

Rat oligodendrocyte precursor cells (OPCs) were prepared from mixed glial cultures as described previously (McCarthy and de Vellis, 1980, Bechler et al., 2015). Briefly, cortices of postnatal day 0 (P0) to P2 Sprague Dawley rats were dissected out. The tissue was digested with 1.2 U/ml papain, 0.1 mg/ml l-cysteine, and 0.40 mg/ml DNase for 1 h at 37°C. Tissue was cultured in DMEM, 10% FCS, and 1% penicillin/streptomycin in T75 flasks; and precoated with 5 µg/ml poly-d-lysine (PDL), at a density of 1.5 brains/flask. Cells were grown at 37°C in 7.5% CO_2_ with medium changes every 2–3 d. After 10–12 d, cells were mechanically separated on an orbital shaker at 250 rpm, 37°C. Loosely attached microglia were removed by shaking for 1 h. Further shaking for 16–18 h detached OPCs. Cell yield was counted using a hemocytometer and plated in assay-dependent conditions, as described below. For patch-clamp electrophysiology, oligodendroglia cultures were transduced with a lentivirus driving EGFP expression using the M1M4 MBP gene promoters (Dib et al., 2011; custom made, Biomolecular Core, University of Edinburgh). Cells were transduced 24 h after plating at least 4 d before recording.

##### Microfiber cultures.

Custom parallel-aligned 1- to 2-μm-diameter poly-l-lactic acid microfibers were synthesized and suspended over plastic scaffolds fitting into 12-well tissue culture plates or were purchased from the Electrospinning Company. Microfibers were washed with 70% EtOH for 10 min followed by coating with PDL for 1 h at 37°C in a 12-well tissue culture plate. Microfibers were washed twice with sterile water and left in preheated myelination media. A total of 35,000 rat OPCs in myelination media [50:50 DMEM/Neurobasal Media, homemade NS21 (Chen et al., 2008), 5 µg/ml *N*-acetyl cysteine, 10 ng/ml d-biotin, ITS (insulin, transferrin, and sodium selenite) supplement, and modified Sato solution (100 µg/ml BSA fraction V, 60 ng/ml progesterone, 16 µg/ml putrecsine, 400 ng/ml tri-iodothyroxine, and 400 ng/ml l-thyroxine; all reagents from Sigma-Aldrich)] were triturated to break up cell clumps and were added dropwise to the microfibers. Cells were left to recover for 3 d before media changing and the addition of treatment, followed by subsequent media changes every 3 d. After 14 d of culture, cells were fixed in 4% PFA for 15 min. To visualize myelination, cells were permeabilized with 0.1% Triton X-100 for 10 min and stained for MBP (1:250) overnight at 4°C followed by incubation with Alexa Fluor 488-conjugated goat anti-rat (1:1000) for 1 h at room temperature and Hoechst stain for 5 min to visualize nuclei. To analyze myelination, individual myelinating oligodendrocytes from one coverslip were imaged (with the experimenter blind to condition) using an SP8 confocal at 40× magnification with a *z*-step of 0.35 µm. The same settings (e.g., laser power, gain, offset) were used between coverslips. ImageJ was used to analyze myelination, again with the experimenter blind to condition. A sheath was defined as a continuous MBP-positive wrap fully surrounding a microfiber, as assessed using the 0.35 µm *z*-series. Concentric tubes were traced, and the length was measured. In addition, the number of concentric sheaths made per individual oligodendrocyte was recorded. Sheath lengths were grouped into 5 µm bins, and the frequency from one experiment was calculated. Mean frequencies from at least three experiments were generated and plotted as a frequency distribution. In some experiments, ZD7288 (catalog #15228, Cayman Chemical) was added to block HCN channel function, as described further below.

##### Mouse oligodendrocyte precursor cell culture.

Mouse OPCs were isolated from P6–P9 pups, as described previously (Watkins et al., 2008; Swire and ffrench-Constant, 2019). Ear clips were taken for subsequent genotyping. Briefly, cerebral cortices were dissected, diced, and dissociated into single-cell suspensions gently using MACS Neural Tissue Dissociation Kit P (catalog #130–092-628, Miltenyi Biotec). Cells were resuspended in 0.2% BSA, insulin, and PBS, and were transferred to treated tissue culture dishes coated with BSL1 (catalog #L-1100, Vector Laboratories) twice for 15 min. Cell solutions were then transferred to dishes coated with anti-PDGFRα (CD140a) for 45 min. Solutions were aspirated, and attached cells were washed twice with media and removed with a cell scraper. All collected cells were added to vented T75 flasks and grown at 37°C with 7.5% CO_2_. Cells were grown in myelination media containing PDGF and neurotrophin-3, were changed every 2 d, and were supplemented daily with PDGF. After 7–9 d, confluent flasks were washed with PBS and then detached using TrypLE for 10 min at 37°C. Solutions were centrifuged at 1000 rpm for 5 min, resuspended, and counted using a hemocytometer.

##### Quantitative PCR.

A total of 75,000 mouse OPCs were cultured on precoated PDL six-well plates for 2 d. RNA was extracted from cells using a RNeasy Mini Kit (Qiagen). RNA was then converted to cDNA libraries using a SuperScript First-Strand Synthesis System. SYBR green quantitative PCR (qPCR) was performed using primers either bought from Qiagen or designed at 0.5 μm. qPCR was performed on a LightCycler 480 II. CT values from designed primers were normalized against GAPDH (QuantiTect Primer Assay-QT01658692, Qiagen).

##### Western blot.

For Western blot analysis, 9 cm treated plastic tissue culture dishes were coated with PDL for either 1 h at 37°C or overnight at room temperature. One million OPCs were added in myelination media to coated plates. Cells were lysed and scraped into RIPA buffer with protease and phosphatase inhibitors (catalog #539134 and #524621, respectively, Calbiochem) for 10 min on ice. Lysates were spun at 16,000 × *g* for 10 min, and supernatants retained. Protein concentration was estimated using a BCA assay kit and loaded into precast protein gels with a protein marker of known molecular weights. Gels were run at 60 V for 30 min and then increased to 100 V for 1 h. Protein was transferred from the gels to nitrocellulose membranes pretreated with methanol at 400 mA for 2 h on ice. Membranes were blocked in 4% BSA in TBS-0.1% Tween (TBST) for 40 min and incubated with primary antibodies [β-actin (catalog #ab8226, Abcam), MBP, CNPase CNPase (catalog #AMAb91072, Atlas)] overnight at 4°C on an orbital shaker. Membranes were washed in TBS-T for 30 min and incubated with species-specific secondary horseradish peroxide antibodies at room temperature for 1 h. Membranes were washed for a further 30 min and incubated with ECL2 for 5 min. Blots were detected using a LI-COR scanner. Subsequent Western blots were performed using the same membranes following removal of bound antibodies through incubation with stripping buffer for 15 min.

##### Patch-clamp electrophysiology.

For electrophysiological experiments, whole-cell recordings were performed at room temperature (21°C) using electrodes (3–5 MΩ) filled with the following (in mm): 155 K-gluconate, 2 MgCl_2_, 10 Na-HEPES, 10 Na-PiCreatine, 2 Mg_2_-ATP, and 0.3 Na_3_-GTP, at pH 7.3 and 300 mOsm. Cells were typically bathed in an extracellular recording solution comprising the following (in mm): 152 NaCl, 2.8 KCl, 10 HEPES, 2 CaCl_2_, 1.5 MgCl_2_, and 10 glucose, at pH 7.3 and 320–330 mOsm. For all recordings, we initialized HCN recordings within 2 min of whole-cell formation to minimize and influence the leaching of intracellular signaling molecules previously indicated to influence HCN properties (Pian et al., 2006). Current recordings made to investigate HCN current activation properties were performed in the presence of BaCl_2_ (1 mm) to block the influence of inwardly rectifying potassium channels. The protocol consisted of holding cells at −44 mV before applying a preconditioning prepulse (3 s) before jumping to −94 mV (1 s) and measuring the HCN “tail” currents activated by the prepulse. To activate HCN channels, the prepulse was sequentially hyperpolarized from −44 mV down to −154 mV. In this protocol, the currents measured during the tail step therefore reflect the channels activated during the preconditioning steps but with a fixed driving force. To determine the leak current (non-HCN-specific current), we then repeated this protocol in the presence of ZD7288 (30 μm). To determine the leak-subtracted (ZD7288-specific, HCN current), we subtracted the leak current from the activation current. Activation curves were generated from the tail currents. Current recordings were typically low-pass filtered online at 2 kHz, digitized at 10 kHz, and recorded to a computer using the WinEDR V2 7.6 Electrophysiology Data Recorder (J. Dempster, Department of Physiology and Pharmacology, University of Strathclyde, Glasgow, UK; www.strath.ac.uk/Departments/PhysPharm/). Our experiments used the HCN blocker ZD7288 (Tocris Bioscience), dissolved in DMSO. ZD7288 has previously been reported to also inhibit T-type calcium channels (Choi et al., 2007; Sanchez-Alonso et al., 2008) and voltage-gated sodium channels (Wu et al., 2012); however, mature oligodendrocytes have little expression of T-type calcium channels or voltage-gated sodium channels (Zhang et al., 2014).

##### Statistics.

All analyses of myelination were performed using ImageJ with the experimenter blind to condition using a filename randomizer macro. Data are presented with standard deviations (SD) to show the variability, or with standard error of the mean (SE) when averaged data were calculated as the mean per animal or per culture was used for each *n*. Statistical analysis was performed using GraphPad Prism software. Activation curves were fitted with a Boltzmann function. Data were tested for normality using a Kolmogorov–Smirnov test. When data were fitted, a normal distribution parametric *t* test and one-way ANOVA, with Tukey's *post hoc* test were used. Where data did not meet normal distribution, nonparametric Mann–Whitney *U* tests and Kruskal–Wallis tests with Dunn's *post hoc* test were used.

## Results

### Oligodendrocytes express functional HCN2-containing ion channels

**Figure 1.**
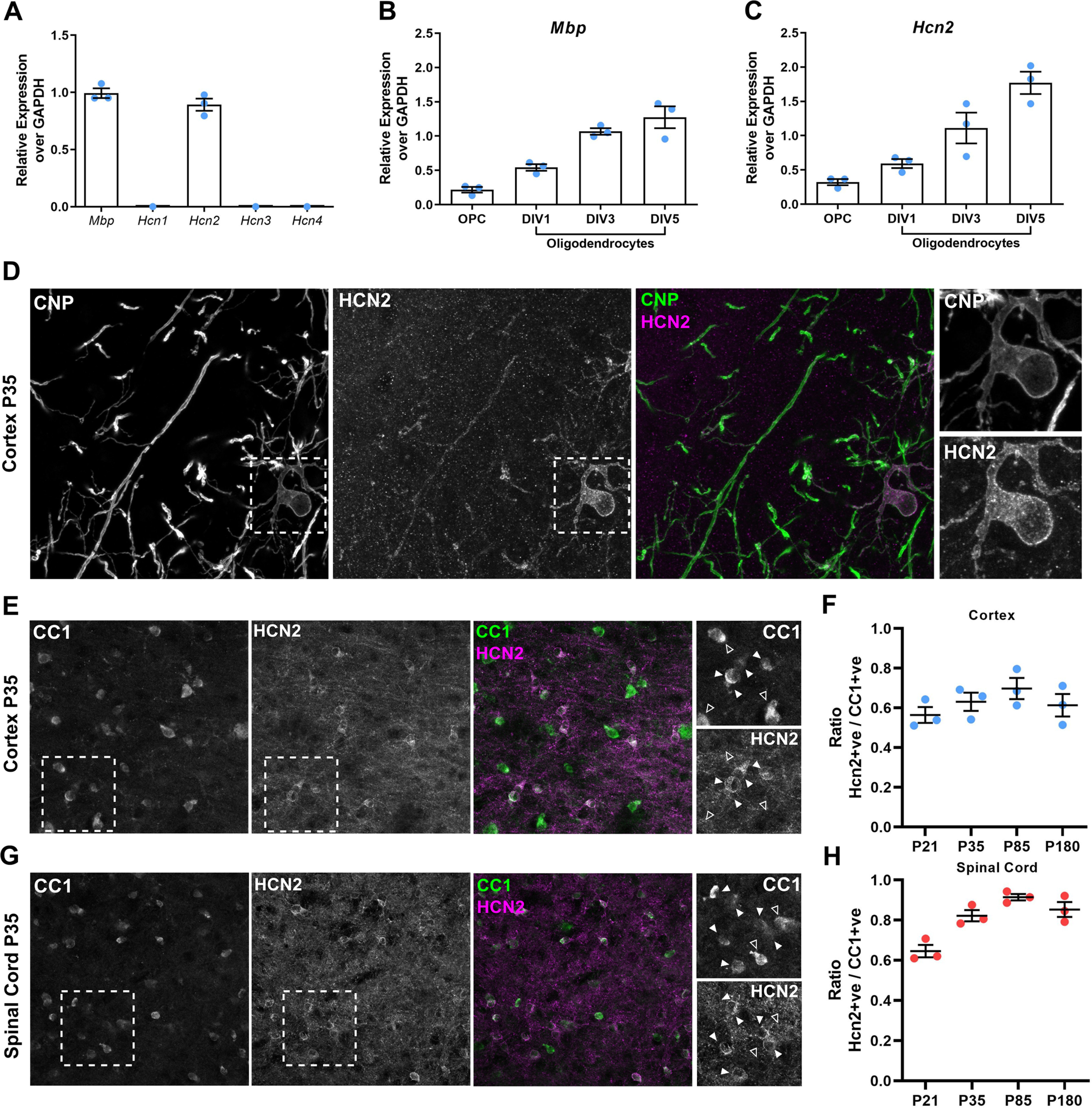
Oligodendrocytes express HCN2 ion channels. ***A***, qPCR of immunopanned mouse oligodendroglia after 5 DIV showing the expression of *Mbp* and *Hcn1-4* normalized to *Gapdh*; *n* = 3 independent cultures. ***B***, ***C***, qPCR of rat oligodendroglial cultures over 7 DIV showing the expression of *Mbp* and *Hcn2* normalized to *Gapdh*; *n* = 3 independent cultures. ***D***, Postnatal day 35 cortical gray matter CNPase-positive oligodendrocyte showing robust HCN2 expression within the cell soma and diffuse staining in myelin sheaths. ***E***, ***G***, HCN2 is expressed by some but not all CC1-positive oligodendrocytes in layer V of the mouse cortex (***E***) and spinal cord (***G***). White arrowheads depict HCN2-positive CC1-positive oligodendrocytes. Empty arrowheads depict HCN2-negative CC1-positive oligodendrocytes. ***F***, Ratio of HCN2-positive CC1-positive oligodendrocytes in the medial prefrontal cortex layer V at P21, P35, P85, and P180 (mean ± SE; P21, 0.5643 ± 0.04; P35, 0.631 ± 0.045; P85, 0.698 ± 0.053; P180, 0.614 ± 0.057); *n* = 3 mice at each time point. ***H***, Ratio of HCN2-positive CC1-positive oligodendrocytes in spinal cord gray matter at P21, P35, P85, and P180 (mean ± SE; P21, 0.646 ± 0.031; P35, 0.822 ± 0.028; P85, 0.915 ± 0.016; P180, 0.853 ± 0.037); *n* = 3 mice at each time point.

HCN ion channels are composed of four isoforms (HCN1–4) and can be assembled using any one of the subunits or any combination thereof. Of the four subunits, HCN2 is the most highly expressed in oligodendrocytes (Zhang et al., 2014; Marques et al., 2016; Lager et al., 2018) and is highly consistent with an almost exclusive HCN2-containing channel population. We confirmed this, identifying only mRNA for *Hcn2* in cultures of immunopanned mouse oligodendrocytes (Fig. 1*A*), also finding increased expression of *Hcn2* with oligodendrocyte maturation (Fig. 1*B*,*C*). We also examined the localization of HCN2 protein *in vivo*, identifying robust expression within oligodendrocyte cell bodies and diffuse staining throughout myelin sheaths (Fig. 1*D*). We further observed that mature cortical oligodendrocyte cell bodies appear to heterogeneously express HCN2 (Figs. 1*D–F*), similar to previous observations in the rat brain (Notomi and Shigemoto, 2004). Analysis of the ratio of HCN2-positive/CC1-positive mature oligodendrocytes in the mouse cortex between P21 and P180 shows a small increase from 55% to 70% by P85, before falling to 62% by P180 (Fig. 1*E*,*F*). In the spinal cord, the vast majority (90%) of oligodendrocytes are HCN2-positive by P85 (Fig. 1*G*,*H*). HCN2-expressing oligodendrocytes are therefore extensive throughout the juvenile and adult CNS and also demonstrate regional variability of expression.

**Figure 2.**
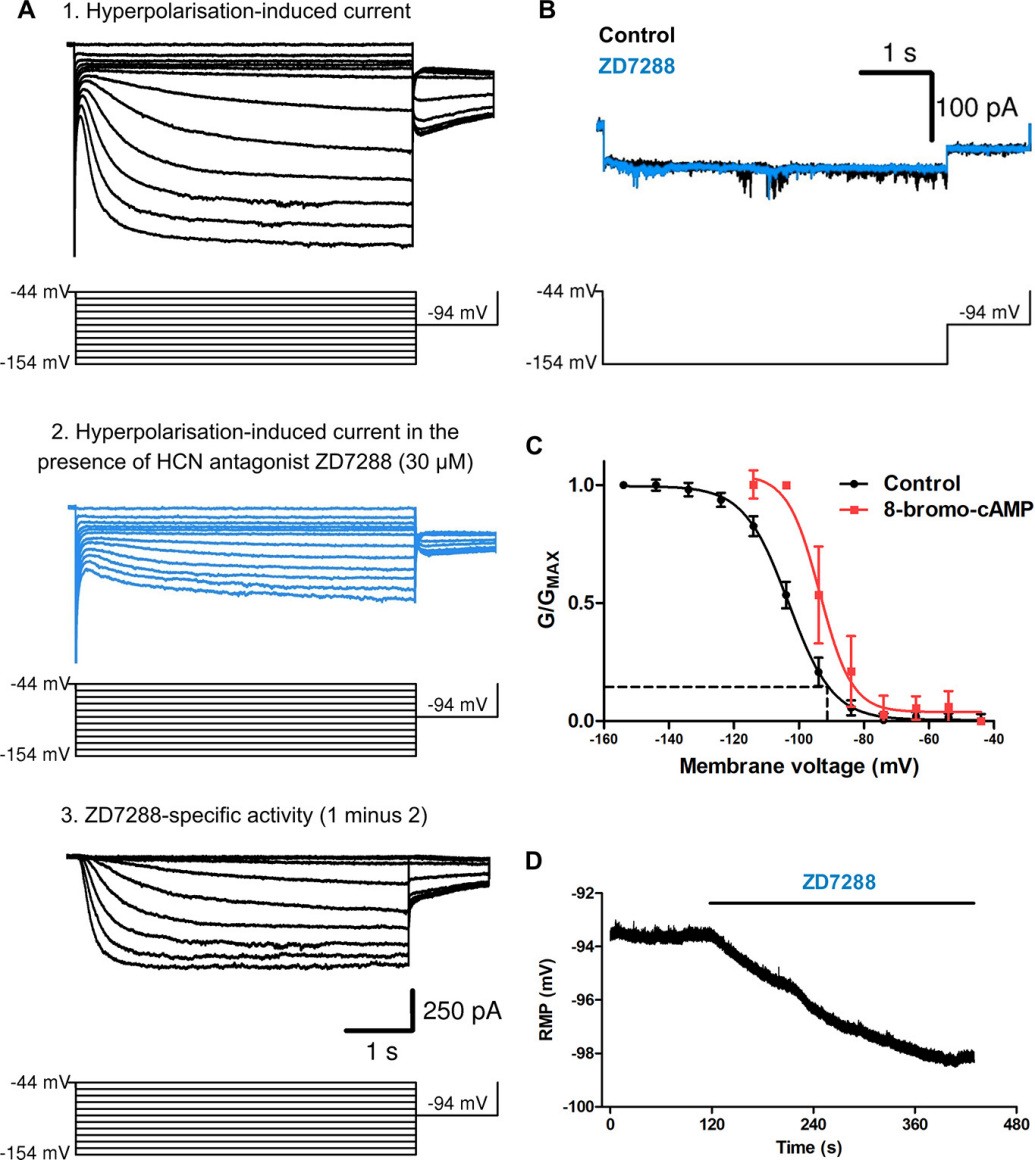
Functional properties of oligodendrocyte HCN channels. ***A***, Whole-cell voltage-clamp currents recorded from an MBP-positive oligodendrocyte in response to an activation protocol (detailed in the bottom panel) in the absence (top) and presence (middle, blue) of HCN channel blocker ZD7288 (30 μm). Bottom, Leak-subtracted currents from the example. HCN currents were measured in the presence of Ba^2+^ (1 mm) in order to block the influence of inwardly rectifying potassium channel currents (see Materials and Methods for full details of the protocol). ***B***, Whole-cell voltage-clamp currents recorded from an MBP-negative oligodendrocyte progenitor in response to an activation protocol (detailed in the bottom panel) in the absence (black) and presence (blue) of HCN channel blocker ZD7288 (30 μm). ***C***, The black plot represents the mean ± SE activation curve of ZD7288-sensitive tail currents (*n* = 6). The mean RMP of oligodendrocytes (dashed line) predicts ∼15% channel opening. The plot in red represents the mean ± SE activation curve of ZD7288-sensitive tail currents in the presence of 8-bromo-cAMP [8-Br-cAMP] (*n* = 6). ***D***, Current-clamp recording from an MBP-positive oligodendrocyte where ZD7288 application generates hyperpolarization of the RMP.

To assess the functional relevance of HCN2-containing channels in oligodendrocytes, we first examined the level of HCN channel activity in oligodendrocytes by performing whole-cell voltage-clamp experiments using cultured rat oligodendrocytes. To visualize and record from differentiated oligodendrocytes for patch-clamp recordings, we transduced cultures with an MBP-GFP reporter. As HCN channels are activated by hyperpolarization, we introduced sequentially more negative hyperpolarizing voltage steps and recorded the current response. To isolate HCN channel currents from other ion channel activity in the oligodendrocyte membrane, we repeated this in the presence of the HCN channel antagonist ZD7288 (Fig. 2*A*). HCN channel-mediated currents were determined by subtracting ZD7288-sensitive currents from those generated by the initial activation protocol without ZD7288. For all MBP-positive oligodendrocytes examined [7 d *in vitro* (7 DIV)], the hyperpolarization-activation protocol induced HCN channel-mediated currents (peak current amplitude, 1009 ± 186 pA; current density, 22.4 ± 5.3 pA/pF; *n* = 11). In contrast, non-MBP EGFP-positive-expressing cells of the oligodendrocyte lineage displayed little, if any, measurable HCN channel activity (Fig. 2*B*). We conclude that these currents were specific to mature oligodendrocytes and were not seen in less mature cells including oligodendrocyte progenitors, in agreement with the transcriptional and proteomic data showing that HCN channels are not expressed at these earlier developmental stages.

### Oligodendrocyte HCN channels are open at normal resting membrane potentials

Having established that oligodendrocytes express functional HCN2 ion channels, we next investigated their physiological significance by determining the influence of HCN2 channels on oligodendrocyte membrane properties. Neuronal HCN channels typically mediate membrane excitability because of the fact that neurons have a hyperpolarized resting RMP sufficient to activate HCN channels (Biel et al., 2009). We confirmed that this is also the case for MBP-positive oligodendrocytes, finding, consistent with previous reports (Larson et al., 2016), these to be highly hyperpolarized (–91 ± 0.8 mV; *n* = 10). To determine the extent to which this hyperpolarized RMP in oligodendrocytes activates HCN channels, we established the voltage-activation profile of oligodendrocyte HCN2 channel-mediated tail currents (Fig. 2*A*) and determined the half-maximal activation potential to be –102 ± 1.4 mV (*n* = 10; Fig. 2*C*). We note that these half-maximal activation properties of HCN-mediated currents in oligodendrocytes are the same as those seen with recombinantly expressed HCN2-containing channels, and are different from neuronal HCN channels that also contain HCN1 subunits, which shift the activation curve to more depolarized potentials (Sartiani et al., 2017). This therefore further supports the conclusion from our expression data that oligodendrocytes express predominately the HCN2 subtype.

From these biophysical data, we can predict that ∼15% of the HCN2 channels in oligodendrocytes are open at RMP, a level comparable to HCN channel activation in neurons (Byczkowicz et al., 2019) and therefore are sufficient to mediate a constant depolarizing conductance. We tested this by performing whole-cell current-clamp experiments to record MBP-positive oligodendrocytes at RMP, and then applied ZD7288 to the bath media. For each oligodendrocyte examined, we observed that ZD7288 generated an increase (−4.3 ± 0.8 mV; *n* = 8) in hyperpolarization of the RMP (Fig. 2*D*), directly demonstrating that HCN2 channels contribute to oligodendrocyte baseline membrane conductance and RMP by generating depolarization. Finally, to test whether oligodendrocyte HCN2 channels were responsive to cAMP, as they are in neurons, we included nonhydrolyzable cyclic nucleotide analog 8-bromo-cAMP to the patch electrode solution and repeated the prepulse protocol described above to measure tail currents. We found that this generated a rightward shift in the activation curve (Fig. 2*C*), increasing the percentage of channels opened at hyperpolarizing voltages, demonstrating that these channels are cyclic nucleotide-gated in MBP-positive oligodendrocytes. The sensitivity to cAMP strongly implicates HCN2 because HCN1 and HCN3 are cAMP insensitive. HCN4, which is also cAMP sensitive, can be ruled out because it has a much slower time constant (Sartiani et al., 2017). Therefore, HCN2-containing channels in oligodendrocytes are hyperpolarization-activated and cyclic nucleotide-gated and thus have the potential to regulate both oligodendrocyte membrane excitability and intracellular signaling.

### Oligodendrocyte HCN currents regulate myelin generation

**Figure 3.**
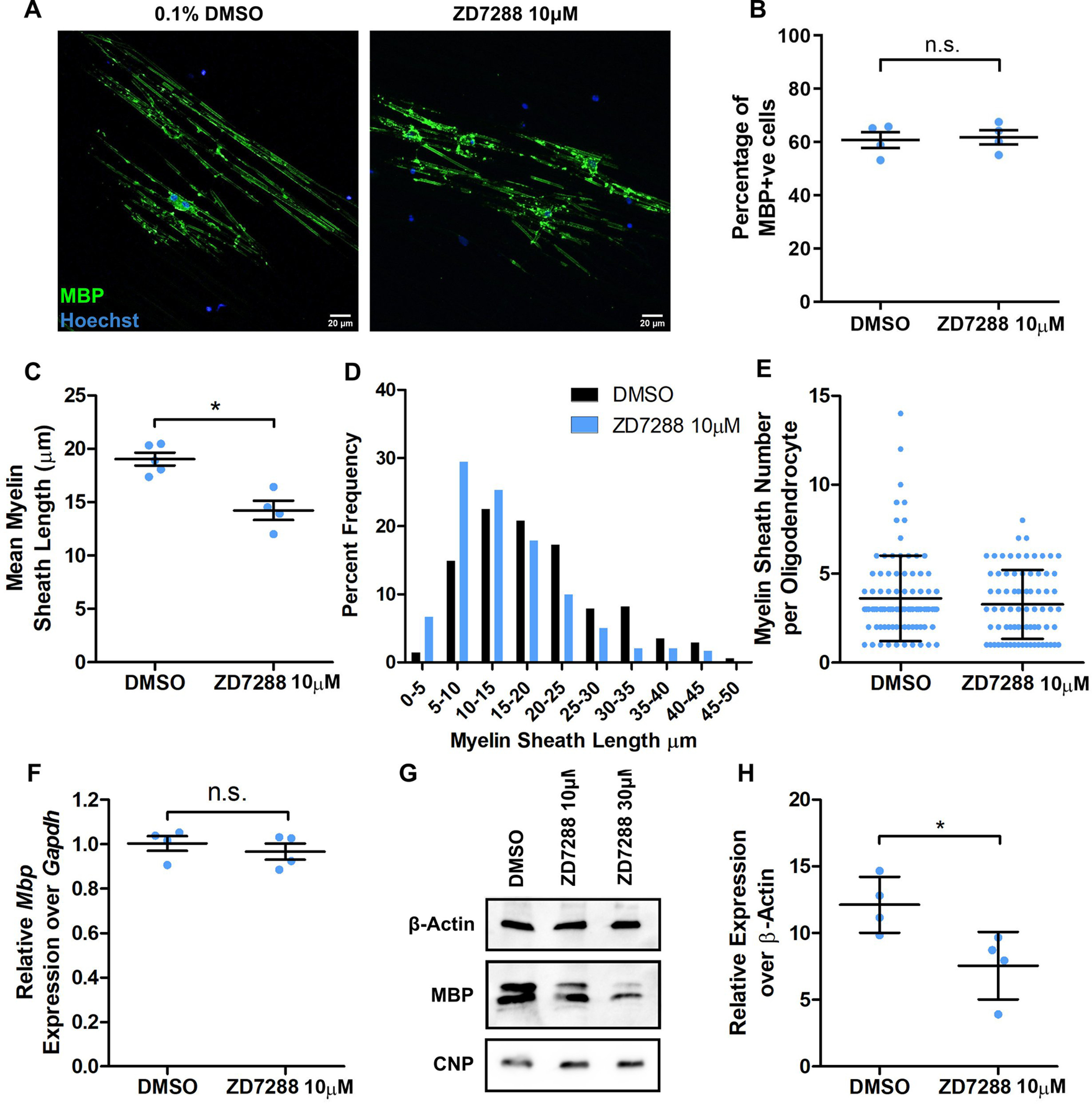
HCN channels regulate the length of myelin sheaths formed in oligodendrocyte-purified cultures. ***A***, Representative MBP-positive oligodendrocytes after 14 d cultured on electrospun microfibers in the presence of either 0.1% DMSO or 10 μm ZD7288. Scale bars, 10 µm. ***B***, Mean ± SE percentage of MBP-positive cells on microfibers: DMSO, 60.77 ± 2.972%; ZD7288, 61.76 ± 2.659%; *n* = 4 independent cultures; *p* = 0.8857, Mann–Whitney test. ***C***, Mean myelin sheath length generated on microfibers. Values are reported as the mean ± SE; DMSO: 19.03 ± 0.6101µm; *n* = 342 sheaths from five independent cultures; ZD7288: 14.23 ± 0.9092 µm; *n* = 241 sheaths from four independent cultures; *p* = 0.0159, Mann–Whitney test. ***D***, Histogram representation of the frequency of sheath lengths generated by oligodendrocytes as assessed by measuring complete MBP-positive sheaths surrounding microfibers in 5 µm bins. Pooled data from *n* = 4–5 independent cultures. ***E***, Pooled data of the number of complete sheaths formed by individual oligodendrocytes in microfiber cultures. Values are reported as the mean ± SD; DMSO: 3.613 ± 2.4; *n* = 93 cells; ZD7288: 3.280 ± 1.935; *n* = 75 cells; pooled data from *n* = 4–5 independent cultures; *p* = 0.5695, Mann–Whitney test. ***F***, qPCR of two-dimensional 3 DIV rat oligodendroglial cultures treated with ZD7288 for the expression of Mbp normalized to Gapdh. DMSO, 1.003 ± 0.03327; ZD7288, 0.9658 ± 0.03675; *n* = 4 independent cultures; *p* = 0.4857, Mann–Whitney test. ***G***, Representative Western blot images for loading control β-actin, MBP, and CNPase from two-dimensional rat oligodendroglial cultures treated with ZD7288. ***H***, MBP protein expression in arbitrary units (A.U.) normalized to β-actin levels. Values are reported as the mean ± SD; DMSO, 12.12 ± 2.084; ZD7288, 7.558 ± 2.540; *n* = 4 independent cultures; *p* = 0.0286, Mann–Whitney test.

To determine whether HCN2 ion channels might influence myelination, we first wanted to assess whether HCN2 channel activity might regulate oligodendrocyte differentiation. We used three-dimensional microfiber cultures in which cortical oligodendrocyte progenitors differentiate and form myelin sheaths of physiological length (Bechler et al., 2015). In these cultures, we blocked HCN2 activity using ZD7288 and found no effect on the percentage of MBP-expressing cells generated (Fig. 3*B*), suggesting that HCN channels do not regulate oligodendrocyte differentiation. To assess whether HCN2 activity might influence myelination directly, we performed two sets of experiments. First, we analyzed the extent of myelin sheath formation in microfiber cultures. Blocking HCN2 channels with ZD7288 caused a significant reduction in the lengths of myelin sheaths formed, without any change in the number of sheaths made by oligodendrocytes (Fig. 3*C–E*). Second, we analyzed transcript and protein levels of the essential myelin protein MBP in cultured oligodendrocytes following treatment with ZD7288. This caused a significant reduction in MBP protein levels yet had no effect on the expression of *Mbp* mRNA (Fig. 3*F–H*). The protein levels of another major myelin component, CNPase, was not altered by ZD7288 (Fig. 3*G*), from which we suggest that HCN2 ion channels may regulate myelin sheath growth by influencing the local translation of key myelin proteins (Muller et al., 2013). Together, these cell culture experiments show that HCN2 ion channels are not required for oligodendrocyte differentiation but regulate the normal levels of myelin generation by differentiated oligodendrocytes, as evidenced by the effects of HCN2 inhibition on the production of essential myelin proteins and smaller myelin sheaths.

### Oligodendrocyte HCN2 regulates myelin sheath length *in vivo*

**Figure 4.**
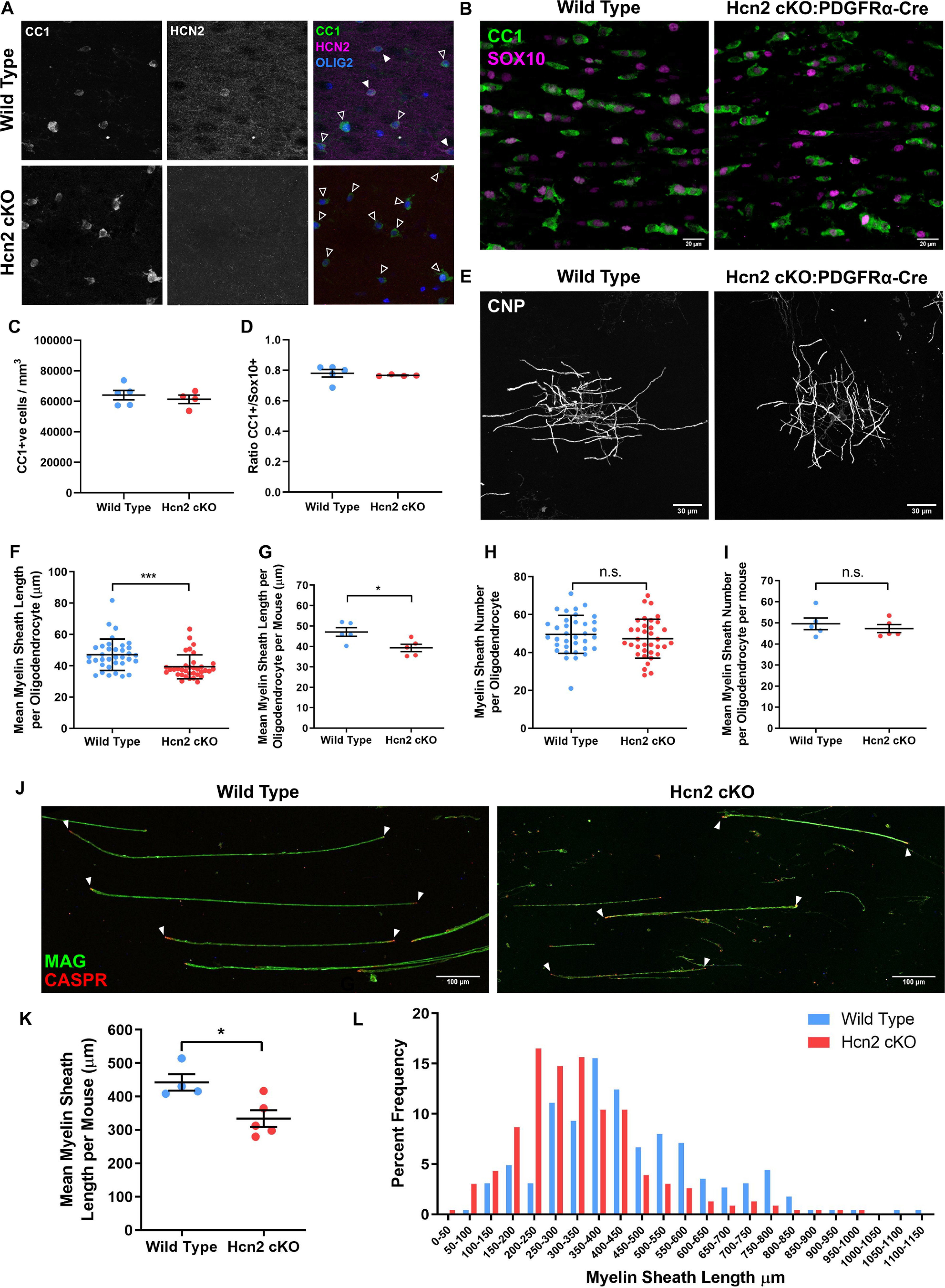
HCN2 regulates the lengths of myelin sheaths formed *in vivo*. ***A***, Postnatal day 21 gray matter oligodendrocyte showing no HCN2 expression in CC1-positive oligodendrocytes in PDGFRa-cre cKO mice. White arrowheads depict HCN2-positive CC1-positive oligodendrocytes. Empty arrowheads depict HCN2-negative CC1-positive oligodendrocytes. ***B***, Representative images of P21 corpus callosum showing expression of CC1 and Sox10. Values are reported as the mean ± SE; wild type: 64,149 ± 3068 cells/mm^3^; Hcn2: 61,386 ± 2749 cells/mm^3^; *n* = 5–4. ***C***, Mean number of CC1-positive oligodendrocytes in the corpus callosum. ***D***, Mean ratio of CC1-positive cells over total Sox10-positive cells. Wild type: 0.7803 ± 0.025; Hcn2: cKO: 0.766 ± 0.003; *n* = 5–4. ***E***, Representative postnatal day 21 gray matter CNPase-positive oligodendrocytes. CNPase staining labels the entire oligodendrocyte enabling the reconstruction of individual cells in the sparsely myelinated layer II/III of the cortex. Scale bars, 30 µm. ***F***, Mean myelin sheath length per oligodendrocyte. Values are reported as the mean ± SD; wild type: 47.08 ± 10.07 µm; *n* = 35 cells from five mice; Hcn2 cKO: 39.34 ± 7.654 µm; *n* = 35 cells from five mice; *p* = 0.0002, Mann–Whitney. ***G***, Mean myelin sheath length per oligodendrocyte per mouse. Wild type: 47.08 ± 2.157 µm; *n* = 1383 sheaths from five mice; Hcn2 cKO: 39.34 ± 1.775 µm; *n* = 1298 sheaths from five mice; *p* = 0.0317, Mann–Whitney test. ***H***, Mean myelin sheath number per oligodendrocyte. Values are reported as the mean ± SD; wild type: 49.57 ± 9.992; *n* = 35 cells from five mice; Hcn2 cKO: 47.29 ± 10.30; *n* = 35 cells from five mice; *p* = 0.3493, unpaired *t* test. ***I***, Mean myelin sheath number per oligodendrocyte per mouse. Values are reported as the mean ± SE: wild-type: 49.57 ± 2.746; *n* = 5; Hcn2 cKO: 47.29 ± 1.856; *n* = 5 mice; *p* = 0.6905, Mann–Whitney test. ***J***, Postnatal day 21 spinal cord white matter teased fibers showing the expression of MAG-positive myelin sheaths and CASPR-positive paranodes. White arrowheads show complete myelin sheaths: two CASPR-positive paranodes connected by a continuous MAG-positive myelin sheath. Scale bars, 100 µm. ***K***, Mean myelin sheath length from spinal cord teased fiber preparations. Values are reported as the mean ± SE; wild-type 442.2 ± 24.39 µm, *n* = 225 sheaths from four mice; Hcn2 cKO: 334.4 ± 24.99 µm; *n* = 230 sheaths from five mice; *p* = 0.0190, unpaired *t* test. ***L***, Histogram representation of data from ***K*** showing the frequency of myelin sheath lengths in 50 µm bins. Pooled data are from *n* = 4–5 mice.

**Figure 5.**
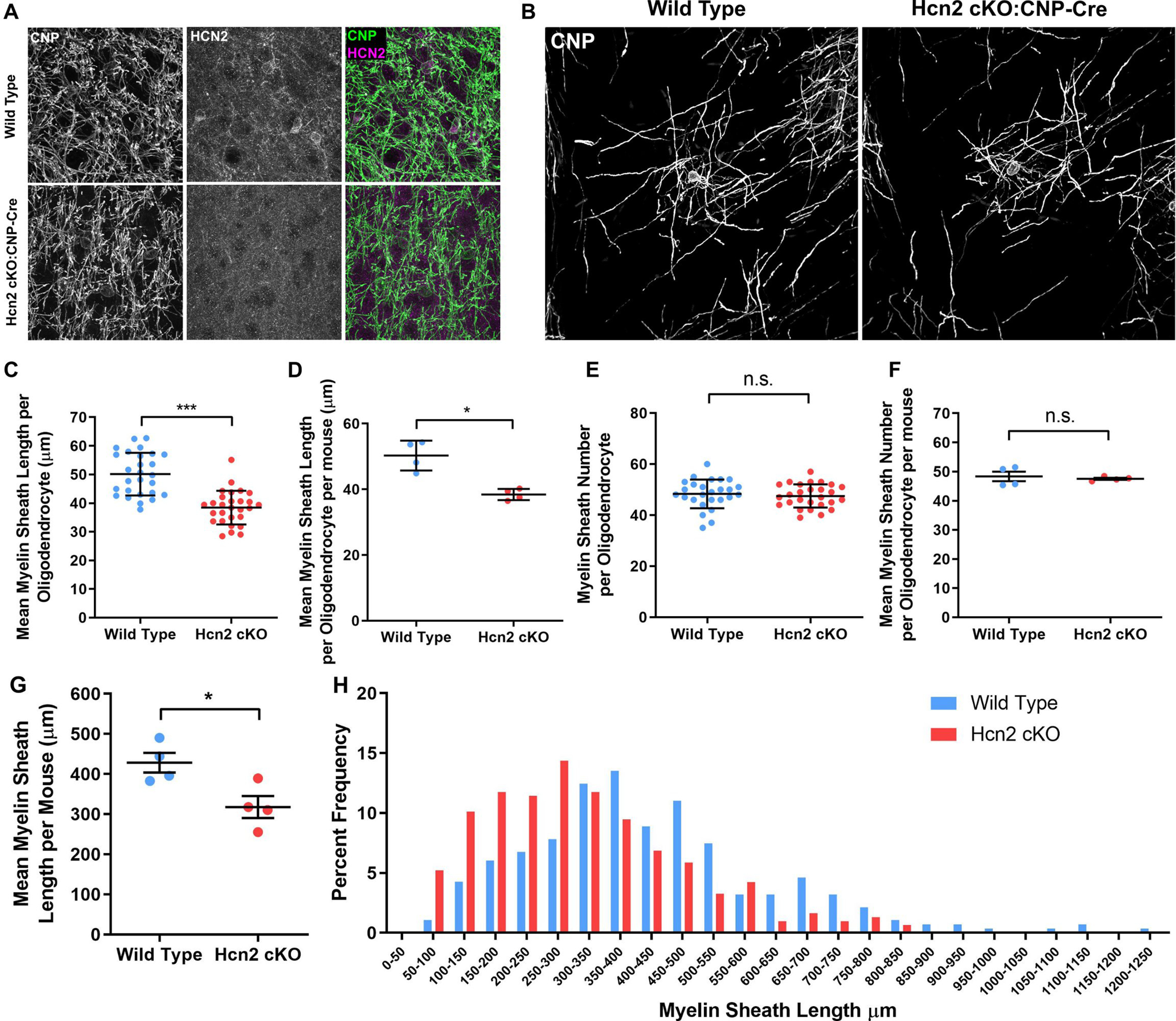
HCN2 regulates the length of myelin sheaths formed *in vivo*. ***A***, Postnatal day 50 gray matter oligodendrocytes showing no HCN2 expression in CNP-positive oligodendrocytes in CNP-cre cKO mice. ***B***, Representative postnatal day 50 gray matter CNPase-positive oligodendrocytes. ***C***, Mean ± SD myelin sheath length per oligodendrocyte. Wild type: 50.12 ± 7.438 µm; *n* = 27 cells from four mice; Hcn2 cKO: 38.42 ± 5.89 µm; *n* = 28 cells from four mice; *p* = 0.0001 unpaired *t* test. ***D***, Mean myelin sheath length per oligodendrocyte per mouse. Values are reported as the mean ± SE; wild-type: 50.27 ± 2.266 µm; *n* = 981 sheaths from four mice; Hcn2 cKO: 38.42 ± 1.711 µm; *n* = 1306 sheaths from four mice, *p* = 0.029, Mann–Whitney test. ***E***, Mean ± SD myelin sheath number per oligodendrocyte. Wild-type 48.35 ± 5.621; *n* = 26 cells from four mice; Hcn2 cKO: 47.52 ± 4.552; *n* = 27 cells from four mice; *p* = 0.5577 unpaired *t* test. ***F***, Mean ± SE myelin sheath number per oligodendrocyte per mouse. Wild-type: 48.35 ± 1.639; *n* = 5; Hcn2 cKO: 47.52 ± 0.36; *n* = 4 mice, *p* = 0.999, Mann–Whitney test. ***G***, Mean ± SE myelin sheath length from spinal cord teased fiber preparations. Wild-type: 428.1 ± 24.52 µm; *n* = 281 sheaths from four mice; Hcn2 cKO: 318 ± 27.51 µm; *n* = 306 sheaths from four mice, *p* = 0.0244, unpaired *t* test. ***H***, Histogram representation of data from ***G*** showing the frequency of myelin sheath lengths in 50 µm bins. Pooled data are from *n* = 4 mice.

The observation that blocking HCN2 ion channels in cell culture reduced myelin sheath length with no effect on number not only shows that these channels are required for normal myelin generation but also raises the possibility that the channels have a physiological role in determining myelin sheath length, a variable critical for efficient rapid axonal conduction. Next, therefore, we tested this by generating cKO mice using a floxed HCN2 line (Emery et al., 2011) crossed with (1) mice expressing cre driven by the *PDGFRA* promoter to delete HCN2 early in the oligodendrocyte lineage and (2) mice expressing cre driven by the *CNP* promoter to delete HCN2 from newly differentiated oligodendrocytes. Confirming the efficacy of this strategy, no expression of HCN2 could be detected in oligodendrocytes in either line of cKO mice *in vivo* (Figs. 4*A*, 5*A*). In addition, *in vitro* electrophysiology experiments using the hyperpolarizing step protocol of −154 from −44 mV to evoke maximal HCN currents showed that HCN channel activity was not observed in cultured MBP-positive oligodendrocytes from *PDGFRA* cKO mice (WT: 362 ± 103 pA, *n* = 5; HCN2 cKO: 0.1 ± 0.04 pA, *n* = 8). This observation supports our expression studies demonstrating that functional oligodendrocyte HCN ion channels are exclusively composed of HCN2 subunits. Furthermore, and again as predicted, we observed a significant (*p* = 0.043, unpaired *t* test) reduction in the resting membrane potential in HCN2 cKO mice (−95.1 ± 1.5 mV, *n* = 8) versus WT mice (−89.8 ± 1.9 mV, *n* = 8).

Having confirmed the efficacy of our cKO strategies, we analyzed myelin sheaths formed by individual oligodendrocytes in the cortex (Figs. 4*F–I*, 5*B–F*) and spinal cord (Figs. 4*J–L*, 5*G*,*H*). This revealed that, as in the cell culture experiments on artificial fibers, myelin sheath lengths were reduced in both strains of HCN2 cKO mice. In contrast, but in keeping with the cell culture experiments, we observed no changes in the number of myelin sheaths generated by oligodendrocytes (Fig. 4*H–I*, 5*E*,*F*) and no change in the generation of oligodendrocytes from OPC, with the number of Sox10-positive and CC1-positive cells, and the ratio of CC1/Sox10-positive cells, being the same between cKO and WT animals (Fig. 4*B–D*).

To exclude the possibility that the effect on sheath length reflects an effect of HCN2 loss on oligodendrocyte homeostasis rather than on the physiological pathways regulating sheath architecture, we measured the intrinsic membrane properties of whole-cell capacitance (HCN2 *PDGFRA* cKO, 66.9 ± 9.9 pF; vs WT, 61.4 ± 7.8 pF; both *n* = 8 cells; *p* = 0.67, unpaired *t* test) and membrane input resistance (derived from a +50 mV pulse; HCN2 cKO, 40.1 ± 4.6 MΩ; vs WT, 49.8 ± 9.0 MΩ; both *n* = 8 cells; *p* = 0.37, unpaired *t* test). No differences between cKO and WT were observed, from which we conclude that HCN2 ion channels are key regulators of oligodendrocyte membrane physiology and myelination, specifically regulating myelin sheath elongation *in vivo*.

## Discussion

The maturation of oligodendrocyte progenitors to myelinating oligodendrocytes involves major changes in membrane physiology, ion channel composition, and generation of distinct biophysical membrane properties (Káradóttir and Attwell, 2007; Bakiri et al., 2009; Larson et al., 2016). Here we show that, as part of these changes, functional HCN2 ion channels become expressed in myelinating oligodendrocytes. A proportion of these HCN2 ion channels are open in the resting hyperpolarized state, and their depolarizing currents contribute to the RMP of the cell. We also show, using both *in vitro* and *in vivo* myelination assays, that these channels regulate myelin sheath elongation. This provides a step change in our understanding of the roles of specific ion channels and transporters in myelinating oligodendrocytes showing that, in addition to mediating key physiological roles in ion and energy homeostasis (Fünfschilling et al., 2012; Lee et al., 2012; Saab et al., 2016; Larson et al., 2018; Schirmer et al., 2018; Suminaite et al., 2019), they can also regulate myelin sheath architecture.

All the evidence in the present study points strongly to an involvement of HCN2 ion channels in the regulation of myelin sheath elongation, rather than one or more of the three other HCN isoforms. First, we found no evidence for the expression of mRNA for any of the other three channels in oligodendrocytes. Second, the activation curve of the current was shifted in a positive direction by elevation of intracellular cAMP, which is only observed for HCN2 and HCN4, but the time constant of activation of the current was relatively rapid, which is not consistent with an involvement of HCN4. Finally, the effects on internodal length of pharmacological block of HCN channels by ZD7288 *in vitro* was fully accounted for, both *in vitro* and *in vivo*, by targeted deletion of HCN2 using two independent Cre drivers.

Our experiments examining HCN2 expression between P21 and P180 reveal that a small minority of oligodendrocytes do not express HCN2 in cortex and spinal cord. We could not distinguish whether this resulted from distinct populations of permanently HCN2-positive or HCN2-negative cells, or as a result of HCN2 only being expressed at specific stages of oligodendrocyte maturation. However, when we compared sheath length between HCN2-positive and HCN2-negative oligodendrocytes in adult wild-type mice, we found no difference (our unpublished observations), rather than the shorter sheaths in the HCN2-negative cells that would be predicted by the cKO experiments if they never expressed this channel. This suggests rather that HCN2 expression occurs in all oligodendrocytes at some stage during their maturation, and the apparent heterogeneity in HCN2 expression reflects different stages of maturation in each part of the CNS.

Our demonstration that oligodendrocyte HCN2 channels regulate myelin sheath length significantly extends our understanding of the biology of these ion channels, previously predominantly implicated in neuronal and cardiac excitability. It also generates the novel hypothesis that HCN2 ion channels might link sheath geometry to extrinsic axonal signals such as those generated by changes in neuronal activity. The conductive and pharmacological properties of HCN2 ion channels suggest two broad mechanisms by which they might respond to extrinsic signals and regulate myelin sheath growth. First, HCN2 ion channels are permeable to Ca^2+^ in addition to sodium and potassium (Yu et al., 2004). Hyperpolarization of oligodendrocytes in the biophysical range of HCN2 ion channel activation could regulate intracellular Ca^2+^ concentration in myelin sheaths (Battefeld et al., 2019), levels of which have previously been associated with developmental changes in myelin sheath growth (Baraban et al., 2018; Krasnow et al., 2018). Second, we have demonstrated that oligodendrocyte HCN2 channels, like neuronal HCN2 channels, respond to intracellular cyclic nucleotide signaling by shifting the activation curve such that HCN2 ion channels will be more likely to be open under typical RMP. Thus, intracellular signaling by cAMP, regulated downstream of many G-protein-coupled and other cell surface receptors, could tune HCN2 activity and thus myelination.

The identification of an oligodendrocyte channel linking the regulation of sheath geometry to extrinsic axonal signals generated by changes in neuronal activity would have important implications for studies exploring the adaptive myelination hypothesis—the proposal that changes in myelination in response to activity can alter circuit function. The discovery provides a potential mechanism for, among others, receptors responding to the release of neurotransmitter from active axons to alter HCN2 activity via intracellular signaling mechanisms and so to mediate localized changes in Ca^2+^ activity that regulate sheath growth. Testing this mechanism and defining the significance of HCN2 ion channel regulation of sheath length for circuit function in the CNS will require further morphologic, electrophysiological, and behavioral analyses of the cKO mice.
